# Surveillance recommendations based on an exploratory analysis of respiratory syncytial virus reports derived from the European Influenza Surveillance System

**DOI:** 10.1186/1471-2334-6-128

**Published:** 2006-08-09

**Authors:** Tamara J Meerhoff, Douglas Fleming, Ann Smith, Anne Mosnier, Arianne B van Gageldonk-Lafeber, W John Paget, the EISS RSV Task Group

**Affiliations:** 1 Netherlands Institute for Health Services Research (NIVEL), EISS-coordination centre, Utrecht, The Netherlands; 2 Royal College of General Practitioners, Birmingham, UK; 3 Health Protection Scotland, Glasgow, UK; 4 GROG/Open Rome, Paris, France; 5 National Institute of Public Health and the Environment, Bilthoven, The Netherlands

## Abstract

**Background:**

Respiratory syncytial virus (RSV) is an important pathogen that can cause severe illness in infants and young children. In this study, we assessed whether data on RSV collected by the European Influenza Surveillance Scheme (EISS) could be used to build an RSV surveillance system in Europe.

**Methods:**

Influenza and RSV data for the 2002–2003 winter season were analysed for England, France, the Netherlands and Scotland. Data from sentinel physician networks and other sources, mainly hospitals, were collected. Respiratory specimens were tested for influenza and RSV mainly by virus culture and polymerase chain reaction amplification.

**Results:**

Data on RSV were entered timely into the EISS database. RSV contributed noticeably to influenza-like illness: in England sentinel RSV detections were common in all age groups, but particularly in young children with 20 (40.8%) of the total number of sentinel swabs testing positive for RSV. Scotland and France also reported the highest percentages of RSV detections in the 0–4 year age group, respectively 10.3% (N = 29) and 12.2% (N = 426). In the Netherlands, RSV was detected in one person aged over 65 years.

**Conclusion:**

We recommend that respiratory specimens collected in influenza surveillance are also tested systematically for RSV and emphasize the use of both community derived data and data from hospitals for RSV surveillance. RSV data from the EISS have been entered in a timely manner and we consider that the EISS model can be used to develop an RSV surveillance system equivalent to the influenza surveillance in Europe.

## Background

Respiratory syncytial virus (RSV) is the most important viral agent causing severe respiratory disease in infants and young children [1]. Although infrequently recognised, RSV infection is common in adults and sometimes causes severe illness especially in the elderly [2,3]. RSV infection presents with similar clinical symptoms to other respiratory viral infections, including influenza [4,5]. Influenza is associated with increased general practice consultation rates [6], increased hospital admissions [7] and excess deaths [7,8]. RSV and influenza viruses frequently co-circulate around the same time of the year making it difficult to estimate their separate clinical impacts [9]. The contribution of RSV to influenza-like illness needs to be assessed if this is to be used as a clinical endpoint for evaluating influenza vaccine effectiveness [10,11].

Advances in the development of RSV vaccines [12] has prompted a need for research into the societal and economic impact of RSV infection in order to make sensible decisions about their potential use. So far, prevention of severe RSV-associated bronchiolitis has only been achieved in high-risk infants by passive administration of the humanized monoclonal antibody palivizumab [13]. A timely RSV surveillance system could be valuable in optimizing the use of palivizumab by increasing its efficiency and reducing costs [14] as doctors would become aware of the circulation of the virus and probable cause of illness in high-risk infants.

Monitoring influenza activity has been coordinated by the European Influenza Surveillance Scheme (EISS) since 1996. EISS is one of the Designated Surveillance Networks established to monitor infectious diseases in the European Union [15]. The surveillance is performed by sentinel primary care physicians and is based on an integrated clinical and virological surveillance model [16,17]. In addition to the sentinel surveillance, results on specimens obtained from other sources (mostly hospitals) are also reported. Currently, no integrated European surveillance such as the EISS is in place for RSV, although RSV surveillance initiatives have been reported from several EU Member States (Germany, the Netherlands, France, United Kingdom).

We aimed to assess whether data already collected within EISS could be used to build an RSV surveillance system in Europe. We consider timeliness of RSV reports to EISS as well as the collection of both sentinel and hospital-based RSV data by age group important for RSV surveillance. We analysed RSV and influenza virus reports in different age groups and study populations in four European countries, and we assessed whether RSV and influenza data were reported in a timely manner into the EISS database.

## Methods

Influenza and RSV data available in the EISS database for the 2002–2003 winter (weeks 40/2002 to 20/2003) were analysed. Data from both sentinel practitioners and other sources (from hospitals, non-sentinel physicians, residential institutions) were used. Data from these other sources are referred to as non-sentinel in this paper. Four countries were included: England, France, the Netherlands and Scotland. Data for France was confined to nine regions in the south covering 37.5% of the French population. The selection of countries was based on the availability of both sentinel and non-sentinel virological data on RSV and influenza, and on a minimum number of 500 non-sentinel respiratory specimens tested for RSV and/or influenza during the study winter.

### Specimen collection and analysis

Combined nose and/or throat swabs were obtained from selected patients presenting to physicians in sentinel practices with influenza-like illness. In addition, general practitioners in Scotland were requested to sample patients with acute respiratory infections in the absence of influenza-like illness. The respiratory specimens were transported to participating laboratories mainly by regular mail [18]. Similar laboratory methods were used in three out of four countries (Table 1); France used enzyme-linked assays including ELISA (enzyme-linked immunosorbent assay) instead of RT-PCR (reverse transcriptase polymerase chain reaction) [19]. Although the sensitivity of ELISA has been reported to be lower than RT-PCR, ELISA is reliable for rapid laboratory diagnosis of influenza in infants and young children; for older patients application of virus isolation or RT-PCR is necessary [20]. Samples were defined positive for RSV or influenza when at least one laboratory test yielded a positive result.

**Table 1:** Laboratory methods used for RSV and influenza virus detection or isolation.

	**Methods used for RSV detection/isolation**	**Methods used for influenza virus detection/isolation**
**England**	RT-PCR, culture	RT-PCR, culture
**France**	ELFA, culture	ELISA, culture
**Netherlands**	RT-PCR, culture	IF, RT-PCR, culture
**Scotland**	RT-PCR (multiplex)	RT-PCR (multiplex)

ELFA: enzyme-linked fluorescent assay (automated qualitative test)

ELISA: enzyme-linked immunosorbent assay

IF: immunofluorescence; this technique was not performed for sentinel samples

RT-PCR: reverse transcriptase polymerase chain reaction

The sentinel networks in England and Scotland did not apply a precise case definition for influenza-like illness. The case definition used in France was: sudden onset of respiratory symptoms with infection context (fever, headaches), in the absence of other diagnosis. The case definition in the Netherlands contained the following criteria: an acute onset of illness (prodromal stage ≤ 3–4 days), and at least one of the symptoms: coughing, rhinitis, sore throat, frontal headache, retrosternal pain, or myalgia [21]. The selection of patients for swabbing was not based on pre-established diagnostic criteria. In France many samples were obtained from children because paediatricians as well as general practitioners are included in the surveillance network [22]. Virological test results from sentinel practices were specified by age group (0 to 4, 5 to 14, 15 to 64, and over 65 years). Non-sentinel specimens obtained from hospitals were mostly examined for one or other and not both of the viruses.

### Data analysis

We examined the timeliness of RSV data entry into the EISS database by investigating whether data on RSV were included in the EISS Weekly Electronic Bulletin and compared this to the timeliness of influenza data. The Weekly Electronic Bulletin is published on the EISS website each Friday and reports the influenza activity for EISS member countries collected during the previous week. More details on the Weekly Electronic Bulletin can be found in the technical note [23]. For the statistical analysis, the comparisons of percentages were performed using EpiTable in Epi Info version 6.04d (January 2001). Statistical significance was concluded if the p-value was < 0.05.

## Results

### Respiratory Syncytial Virus

RSV detections are summarized for each of the four countries in Table 2. For England RSV detections from sentinel practices were common in all age groups, but especially in young children aged 0–4 years with 40.8% (N = 49) testing positive for RSV. The highest percentage RSV positive specimens was reported for the 0–4 age group in Scotland (10.3%, N = 29) and France (12.2%, N = 426) as well. In the Netherlands, RSV was detected in one person aged over 65 years. In England, the percentage of RSV positive reports (26.7%) was higher than that for influenza (21.3%; Chi^2 ^= 3.9, p = 0.048). Non-sentinel data (available by age for England and Scotland only) showed that 92% or more of the RSV positive reports were obtained in children 0 to 4 years.

**Table 2:** RSV detections by country and age group for sentinel and non-sentinel specimens.

		***Sentinel***			***Non-sentinel***
***Country***	**Age group (years)**	**Total number of specimens tested**	**Number of specimens tested positive for RSV**	**Percentage positive (%)**	**Number of specimens tested positive for RSV**

England	0–4	49	20	40.8	3,982
	5–14	63	16	25.4	13
	15–64	307	77	25.1	60
	> 65	45	13	28.9	11
	NK	11	1	9.1	85
	**Total**	**475**	**127**	**26.7**	**4,151**
Scotland	0–4	29	3	10.3	1,474
	5–14	67	0	-	24
	15–64	444	13	2.9	56
	> 65	58	3	5.1	19
	NK				15
	**Total**	**598**	**19**	**3.2**	**1,588**
France	0–4	426	52	12.2	
	5–14	442	20	4.5	
	15–64	557	14	2.5	
	> 65	32	0	-	
	**Total**	**1,457**	**86**	**5.9**	**1,748**
Netherlands	0–4	0	-	**-**	
	5–14	7	0	-	
	15–64	42	0	-	
	> 65	7	1	14.3	
	**Total**	**56**	**1**	**1.8**	**1,757**

NK: not known

### Influenza

Influenza virus detections are summarized in Table 3. Sentinel data indicated more influenza reports than RSV in Scotland, France and the Netherlands. The highest specimen positive proportions of influenza viruses were reported in children aged 5–14 years (England 52.4%; France 41.6%; Scotland 23.9%). Non-sentinel data (available by age for England and Scotland only) showed most confirmed influenza cases in the 0–4 and 15–64 age groups.

**Table 3:** Influenza virus detections by country and age group for sentinel and non-sentinel specimens.

		***Sentinel***			***Non-sentinel***
***Country***	**Age group (years)**	**Total number of specimens tested**	**Number of specimens tested positive for influenza**	**Percentage positive (%)**	**Number of specimens tested positive for influenza**

England	0–4	49	12	24.5	260
	5–14	63	33	52.4	81
	15–64	307	51	16.6	143
	> 65	45	2	4.4	45
	NK	11	3	27.3	12
	**Total**	**475**	**101**	**21.3**	**541**
Scotland	0–4	29	0	0	64
	5–14	67	16	23.9	53
	15–64	444	13	2.9	108
	> 65	58	2	3.4	31
	NK				1
	**Total**	**598**	**31**	**5.2**	**257**
France	0–4	426	82	19.2	
	5–14	442	184	41.6	
	15–64	557	109	19.6	
	> 65	32	1	3.1	
	**Total**	**1,457**	**376**	**25.8**	**243**
Netherlands	0–4	0	0		
	5–14	7	3	42.9	
	15–64	42	8	19.0	
	> 65	7	4	57.1	
	**Total**	**56**	**15**	**26.8**	**239**

NK: not known

### Timeliness

In each of the four countries sentinel and/or non-sentinel RSV data were entered in a timely manner, within 1–2 weeks after specimen collection, into the EISS database. A total of 26 Weekly Electronic Bulletins were published during the 2002–2003 winter season, from week 42 to week 15 of the following year. For the Netherlands, timely RSV data were not available for weeks 42–50 because data entry only started that season. For the four countries data on influenza was reported for a total of 97 out of 104 weeks and RSV reports where made in a timely manner in 87 out of 104 weeks.

## Discussion

We have assessed whether EISS could be used to build a European RSV surveillance system. Surveillance systems must be timely in order to be effective. The EISS system has demonstrated timeliness in providing data on influenza and as this report shows in four countries, timely data on RSV. Sentinel data indicated that RSV contributed considerably to influenza-like illness, especially in young children. Since the infrastructure of EISS is well established [24], we suggest the use of EISS as a model for setting up an RSV surveillance system in Europe.

Healthcare based surveillance systems are dependent upon persons consulting doctors. For common respiratory infections, there are many more infected persons in the community who do not consult their doctor. Selection biases which start with the decision to consult are compounded at the point of consultation. In addition, sensible use of virological investigation does not necessarily mean that every suspect case is investigated. Certainly as far as patients in the community are concerned, routine virological investigation for a common condition which is usually minor is not economically justifiable. Furthermore, the patient's willingness to be sampled will always be a major consideration.

The EISS differentiates between sentinel and non-sentinel sources of data. Sentinel networks in Europe are chiefly based on general practices (and in some European countries also on paediatric primary care services) and these are essential to provide insight into what is happening in the community at large. However, the hospital admission is a useful proxy for severity of illness and it is desirable therefore to have access to additional hospital source data. This is particularly important when an illness is common in all age groups but hospital admission is much more likely in particular age groups. Accordingly we wish to encourage data collection from hospitals either on a routine basis from all hospitals or perhaps more thought might be given to the development of sentinel hospitals with a higher level of commitment to high quality data capture and more structured virological investigation.

Our study has shown that the age distribution of RSV positive cases was similar in the four countries. For England relatively more RSV than influenza was reported but this was not so in the other three countries. A possible reason for this could be the use of a more sensitive diagnostic test in England compared to the other countries. Within EISS the need for harmonization of laboratory methods is recognised and a Community Network of Reference Laboratories has been established in 2003. This Network encourages the harmonisation of laboratory methods for the detection of influenza in EISS and assesses the quality of laboratory testing for influenza and RSV [25].

To see whether the data for England were consistent with earlier findings, we compared our results to data published previously on RSV [10]. More RSV than influenza virus was reported for one of the winter seasons (1997–98), this finding is similar to what we have reported for 2002–2003. It is important to note that differences between countries and seasons can simply be due to seasonal variation; lower proportions of RSV detections from patients with influenza-like illness have been observed for England as well [26].

The sentinel networks in all four countries used combined nose and/or throat swabs inserted in the same vial. These have proved reliable for influenza surveillance [27]. However, the best site to collect material for viral detection may differ between influenza virus and RSV. Nasal swabs may be less specific than nasopharyngeal aspiration [28], on the other hand swabs are probably less painful and easier to obtain in a general practice setting. Facilities for sampling patients in the hospital are generally better than those in the community since there may be increased opportunity for sample collection and less limitation on sample transportation with hospitals linked directly to microbiology laboratories.

The diagnosis made, the selection of patients for swabbing, the quality of the swab taken, the transport procedures, the virological investigation methods and the experience of the laboratory concerned, all influence virus detection rates. The majority of sentinel respiratory specimens did not test positive for either influenza or RSV. This may be explained by other respiratory viruses that are known to cause symptoms similar to influenza and RSV infection [29,30] but few are regularly investigated. As an example, for Scotland, 83 (13.9%) sentinel swabs tested positive for picornavirus during the 2002–2003 winter season. Furthermore, positivity rates differed considerably between countries: e.g. in Scotland the percentage positive for RSV and influenza was only 8%. In the future, the EISS might implement more respiratory viruses for surveillance purposes simultaneously after introducing RSV. Nine countries in EISS already tested sentinel specimens for more viruses than RSV and influenza in 2002, e.g. for human metapneumovirus, rhinovirus, coronavirus, adenovirus, C. pneumoniae or para-influenza virus.

Discrepancies in positivity rates could reflect several factors mentioned above; but it is also possible that payment to general practitioners for taking swabs in Scotland leads to sampling bias. In addition, general practitioners in Scotland are requested to take samples from patients with acute respiratory infections in the absence of influenza. Relatively few respiratory specimens were collected by the Dutch sentinel network which can lead to underestimation of the incidence of RSV and influenza as judged from virological data. This seems in particular true for children and the elderly.

The current methodological differences between countries and the constraints of the study (data for four countries and one season) imposes limitations. Since we selected and analysed data for the four countries that tested sentinel specimens during the 2002–2003 winter season for RSV, we cannot state that all members of EISS are able to comply to routine RSV reporting. However, this study demonstrated that it is possible to report RSV in addition to influenza. We believe our results pave the way for the development of an RSV surveillance system running in parallel to influenza surveillance.

## Conclusion

Our conclusions relate to recommendations for an RSV surveillance programme.

1. Specimens collected as part of an influenza surveillance programme should also be tested for RSV.

2. Both combined nose/throat swabs and nasal pharyngeal aspirates are acceptable for RSV diagnosis.

3. The application of molecular techniques such as real time PCR in the diagnosis of respiratory disease has been demonstrated and we advocate this technique for RSV detection.

4. We encourage further developments on the use of standardized methods and laboratory techniques.

5. The development of a sentinel approach of representative hospitals should be considered.

6. We recommend the new networks joining EISS to integrate RSV surveillance alongside influenza.

## Competing interests

The author(s) declare that they have no competing interests.

## Authors' contributions

TJM carried out the analysis, participated in the design and drafted the manuscript. DF participated in the study design and helped to draft the manuscript. AM, AS and RVGL equally contributed to the study by providing country specific data and helping in the interpretation of data. WJP participated in the design of the study and coordination and helped to draft the manuscript. All authors read and approved the final manuscript.

## Pre-publication history

The pre-publication history for this paper can be accessed here:


